# Patterns and Characteristics of Mobile App Use to Promote Wellness and Manage Illness: Cross-Sectional Study

**DOI:** 10.2196/71363

**Published:** 2026-01-30

**Authors:** Hayriye Gulec, David Smahel, Yi Huang

**Affiliations:** 1 Interdisciplinary Research Team on Internet and Society Faculty of Social Studies Masaryk University Brno Czech Republic; 2 Department of Psychology Faculty of Arts and Sciences Bursa Uludag University Bursa Turkey; 3 Centre for Research and Development Pan-European University Bratislava Slovakia

**Keywords:** mobile health, mHealth, eHealth literacy, eating disorder, ESEM, exploratory structural equation modeling, SNS, social networking site

## Abstract

**Background:**

Mobile health (mHealth) apps target diverse health behaviors, but engagement may vary by purpose.

**Objective:**

This study examined the prevalence, usage patterns, and user characteristics of mHealth apps among Czech adults with internet access, focusing on sociodemographics, digital knowledge and use, and health indicators predicting wellness- and illness-related app use.

**Methods:**

Overall, 4775 Czech adults (2365/4775, 49.53% women) aged 18-95 (mean 45.37, SD 16.40) years completed an online survey. Sociodemographic factors included age, gender, education, and income. Digital knowledge and use were measured using the eHealth Literacy Scale and the passive/active use of social networking sites (SNS) for health information. Health indicators covered symptom severity, physical activity, BMI, and eating disorder–related risk propensity (body dissatisfaction, dietary restraint, and weight/shape overvaluation). Participants reported app use for sports, number of steps, nutrition, vitals, sleep, diagnosed conditions, reproductive health, diagnosis assistance, mood and mental well-being, and emergency care guidance. Multivariate hierarchical binary logistic regression analysis identified characteristics of app users. Exploratory structural equation modeling (ESEM) clustered apps into “promoting wellness” and “managing illness” and examined the predictors of frequency of use.

**Results:**

Of 4440 respondents, 2172 (48.92%) used mHealth apps. Users were younger (odds ratio [OR] 0.98, 95% CI 0.98-0.99, *P*<.001), had a monthly income more than 50,000 CZK (1 CZK=US $0.048; vs ≤20,000 CZK: OR 0.54, 95% CI 0.41-0.7, *P*<.001; 20,001-35,000 CZK: OR 0.78, 95% CI 0.65-0.93, *P*=.006; 35,001-50,000 CZK: OR 0.83, 95% CI 0.7-0.99, *P*=.03), were more eHealth literate (OR 1.17, 95% CI 1.06-1.3, *P*=.003), used SNS passively for health information (OR 1.35, 95% CI 1.21-1.51, *P*<.001), and had higher eating disorder risk (OR 1.18, 95% CI 1.12-1.25, *P*<.001) and physical activity (OR 1.18, 95% CI 1.13-1.23, *P*<.001) than nonusers. Step-counting apps were most common; 65.99% (1430/2167) used them daily or several times a day, followed by apps for sleep (691/2163, 31.95%), vitals (611/2165, 28.22%), and sports (407/2158, 18.86%). ESEM confirmed a 2-factor structure (“promoting wellness” and “managing illness”; *χ*²_26_=71.9, comparative fit index=0.99, Tucker-Lewis index=0.99, root-mean-square error of approximation=0.03, and standardized root-mean-square residual=0.03). Frequent use of wellness apps was associated with younger age (standardized β=–0.22, *P*<.001), higher eHealth literacy (standardized β=0.10, *P*<.001), and physical activity (standardized β=0.15, *P*<.001). Illness-management app use was associated with active use of SNS for health information (standardized β=0.62, *P*<.001) and eating disorder risk (standardized β=0.11, *P*<.001). Digital knowledge, digital use, and health indicators mediated the association between age and mHealth app use.

**Conclusions:**

mHealth app engagement reflects broader social, digital, and psychological inequalities rather than individual preferences alone. Encouraging digital inclusion and addressing body image- and diet-related use may help ensure that mHealth technologies do not exacerbate existing health inequalities across age and user groups.

## Introduction

### Background

The rapid advancements in mobile health (mHealth) app technologies and the continued improvement to content have resulted in the proliferation of health-related areas addressed via mHealth apps and wearables in the last decade [1-3]. Now, smartphone users can monitor and regulate daily routines (eg, number of steps and circadian rhythms) and autonomic indicators (eg, heart rate, blood pressure, body temperature, and respiratory rate). They can use apps to modify lifestyle behaviors (eg, physical activity, nutritional intake, and weight management), improve mental health and well-being (eg, meditation and mindfulness), get health assistance (eg, diagnosis assistance, reproductive health, and emergency care guidance), and manage diagnosed conditions (eg, diabetes and depression).

Two users who download the same app to track and regulate a particular health behavior (eg, nutritional intake) may use it for separate purposes: one to maintain a healthy lifestyle and the other to manage a chronic health condition (eg, diabetes and obesity). The characteristics of users might differ depending on their purpose of use. Although previous literature has linked mHealth app usage to user intentions related to self-management of wellness and illness [4-7], empirical studies have primarily focused on app adoption in general, overlooking user characteristics associated with the purpose of use [8]. Additionally, only a few studies have examined the prevalence and characteristics of mHealth app usage in large-scale, representative samples [9-12].

Therefore, the aim of this study is 2-fold. First, we examine the prevalence and characteristics of app users within a representative sample of Czech adults with internet access. We focus on sociodemographics, digital knowledge and use, and health indicators as user characteristics. Second, we cluster mHealth apps using a data-driven approach under the “promoting wellness” and “managing illness” dimensions. Finally, we determine the user characteristics that predict the app usage frequency for each dimension. This study reports on a large representative adult sample concerning the prevalence, usage patterns, and user characteristics of various types of apps. It also provides initial evidence for user characteristics as predictors of wellness- and illness-related app usage.

Prior large-scale studies examining adult mHealth app users were primarily conducted in the United States using different waves of data from the National Cancer Institute’s Health Information National Trends Survey. The prevalence of mHealth app use among smartphone owners ranged between 34% and 56%, and they consistently indicated younger age and higher educational status as significant predictors [9-12]. Females were more likely to own and use mHealth apps in general [10-12], although no gender difference was reported in one study [9]. A recent scoping review identified age, gender, educational level, and income status as significant factors [8]. This study examines the prevalence and sociodemographic characteristics of mHealth app use among a representative sample of Czech adults with internet access. It also reveals whether these characteristics differ when using apps to promote wellness and manage illness.

eHealth literacy is the perceived knowledge and skills to obtain, understand, and evaluate digital health information [13]. Previous studies reported eHealth literacy as a significant predictor of mHealth app use in general adult samples [14,15] and individuals with chronic health conditions [16,17]. In addition, higher eHealth literacy was associated with the perception of mHealth apps as effective and their frequent usage for health-related behavioral change [18]. This study provides initial insights into whether app usage to promote wellness and manage illness differs as a function of eHealth literacy.

Digital platforms, including social networking sites (SNS; eg, Facebook, Instagram, and X, formerly known as Twitter), are frequently used for health-related purposes [19,20]. Individuals who use social media for health information are more likely to seek health information through mHealth apps, indicating that both media might be used complementarily [21]. Moreover, prior research has shown that health messages delivered via SNS can shape users’ health-related decisions and behaviors [21,22]. Therefore, a positive relationship between SNS activity and the use of mobile apps for health purposes is plausible. This study aims to provide initial evidence on whether health-related SNS use is linked to the adoption of health apps and the frequency of their use for promoting wellness and managing illness.

Prior studies have examined general health status as a predictor of mHealth app use; however, these studies have assessed health status using a single item, yielding mixed findings [9-12]. This study adopts a more nuanced approach to address this limitation, assessing perceived somatic-symptom severity across multiple domains, including gastrointestinal, cardiopulmonary, musculoskeletal, and sleep/energy areas. In parallel, an active lifestyle—as reflected in physical activity levels—has received growing attention in mHealth research, with evidence suggesting that app use may support health promotion among physically active individuals [10,18,23]. By jointly examining both physical activity and detailed health status indicators, this study contributes to understanding how these factors shape the adoption and use of apps for promoting wellness and managing illness.

Lastly, only a few studies examined BMI and eating disorder–related risk factors as correlates of mHealth app use. This study focused on BMI because it is a significant predictor of health outcomes, including diabetes and obesity [24]. Evidence suggests that individuals with a higher BMI are more likely to use mHealth apps [10,18,25] and to use them for a health behavior goal [9,26]. A higher BMI is also a significant predictor of eating disorder symptoms, including body dissatisfaction, dietary restraint, and weight and shape overvaluation [27]. Previous research has reported a connection between using mHealth apps to track diet and physical activity and their associations with eating disorder–related risks and symptoms, including BMI [28-30]. These findings underscore the importance of evaluating core risk factors for eating disorders together with BMI to understand how they might play roles in using apps that address behaviors related to diet and physical exercise (ie, health promotion) but also those related to monitoring health (eg, vitals) and managing disease (eg, obesity and diabetes). In this study, we evaluate body dissatisfaction, dietary restraint, and weight and shape overvaluation to determine participants’ risk propensity for an eating disorder.

### This Study

This study investigated the prevalence and frequency of mHealth app use in a representative sample of Czech adults with internet access. Our first objective was to identify user characteristics that predict app adoption and usage. We examined a comprehensive set of predictors, including sociodemographic variables (age, gender, education, and income), digital knowledge and use (eHealth literacy and SNS use for health information), and health indicators (somatic symptom severity, physical activity, BMI, and eating disorder risk propensity). This multidimensional approach enabled us to move beyond basic demographic correlates and gain a deeper understanding of the factors influencing engagement with mHealth apps. Our second objective was to cluster mHealth apps into two key dimensions—promoting wellness and managing illness—as suggested by the prior literature [4-7]. Rather than relying solely on predefined app classifications, we used a data-driven approach to cluster mHealth app usage into categories related to wellness and illness. Furthermore, we determined user characteristics associated with the frequency of use within each category. This approach provided a more nuanced understanding of user characteristics connected with using wellness and illness app types.

During the analyses, we observed that the effect size of age on app use was consistently reduced after entering the variables related to digital knowledge, digital use, and health indicators. This finding prompted us to conduct additional exploratory mediation analyses to test whether these factors mediated the association between age and app use. These exploratory analyses provide insight into potential mechanisms underlying age-related differences in mHealth engagement, informing future strategies to reduce disparities in app adoption and use for wellness promotion and illness management.

## Methods

### Recruitment

This study used cross-sectional data collected through an online survey targeting adult Czech internet users. The study design adhered to the STROBE (Strengthening the Reporting of Observational Studies in Epidemiology) guidelines for observational cross-sectional research. Eligible participants were adults aged 18 years or older who reported using the internet. Data collection was conducted by STEM/MARK, a Czech market research agency that manages the Czech National Panel (part of National Sample s.r.o.). STEM/MARK is a member of the European Society for Opinion and Market Research and follows its data protection and panel management standards. Data collection occurred between October 2 and 16, 2023, as part of the NPO “Systemic Risk Institute” project.

The Czech National Panel includes 64,000 individuals and is designed to capture a broad spectrum of the population, including harder-to-reach subgroups. Eligible participants were selected from an initial pool of 58,000 participants. A quota-sampling strategy was applied with respect to age, educational attainment, household income, municipality size, and administrative region, following the Nomenclature of Territorial Units for Statistics (NUTS3) classification, as per Eurostat data. The gender distribution was balanced, with a tolerance of ±7% based on the registered gender within the panel. Our recruitment goal was to maximize sample size within the project’s funding constraints, with a minimum target of 3500 respondents to ensure meaningful representation even from the smallest regions.

Participants were recruited through online and face-to-face invitations, and the questionnaire was delivered via computer-assisted web interviewing. The survey was opened by 5480 panelists. Of these, 5135 filled in the items to verify the respondent’s suitability for data collection (eg, quotas). Due to noncompliance with research requirements or failure to fulfill quotas, 214 respondents were rejected. The number of panelists who completed the questionnaire was 4921 individuals. An additional 146 questionnaires were discarded due to excessive missing data (>10%), unrealistically fast completion times, or logically inconsistent answers. The final sample consisted of 4775 adult Czech internet users (2365/4775, 49.53% women) aged 18-95 years (mean 45.37, SD 16.40 years).

All survey instruments underwent cognitive pretesting before data collection. Feedback was obtained from 5 individuals representing diverse genders, ages, and educational backgrounds (aged 26-73 years). Their input focused on evaluating the clarity, comprehensibility, and overall content of the questionnaire.

### Measures

#### Sociodemographic Characteristics

Participants reported their gender and responded to an open-ended question about their age. They indicated their highest education with the following response options: (1) no education or primary school, (2) secondary vocational school, (3) secondary school, and (4) university (or higher vocational school). The income status was assessed by asking, “In which category would you put the average monthly income of your household for all its members? Please consider the income from employment, part-time jobs, business, and social security.” The response options were (1) up to 20,000 CZK, (2) 20,001-35,000 CZK, (3) 35,001-50,000 CZK, and (4) 50,001 CZK and more. [A currency exchange rate of CZK 1=US $0.048 is applicable.] Higher scores were indicative of better financial status.

#### Digital Knowledge and Use Factors

##### eHealth Literacy

eHealth literacy was assessed by the eHealth Literacy Scale [31]. The items inquired about knowledge of online health information sources (eg, I know what health resources are available on the internet), how to navigate the internet to obtain answers to health-related questions (eg, I know where to find helpful health resources on the internet), the perceived skills to evaluate the quality of online health information (eg, I can tell high-quality health resources from low-quality health resources on the internet), and ability to apply health information for health purposes (eg, I know how to use the health information I find on the internet to help me). The response options ranged from 1 (strongly disagree) to 5 (strongly agree). Higher scores indicated better eHealth literacy skills. The internal consistency was adequate (Cronbach α=0.89).

##### Health-Related SNS Use

The health-related use of SNS was assessed using items developed in a previous study to measure both active and passive social media use [32]. We adapted the items to tap into passive and active use of SNS for health information. Passive health-related SNS use refers to the passive engagement with health information. Participants responded to how often they “watched videos or pictures posted about health or illness,” “followed health- or illness-related pages,” and “read health- or illness-related discussions, user comments, ratings, or reviews” on SNS. Active health-related SNS use items evaluated active engagement with the health information and asked how often participants “posted health- or illness-related content,” “chatted or interacted with others about health or illness,” and “commented on, liked, or shared others’ health- or illness-related posts” on SNS. The response options were (1) never, (2) a few times at most, (3) several times a month, (4) several times a week, (5) every day, and (6) several times a day. The scores were calculated separately for passive and active use; the Cronbach alphas were 0.9 for both scales.

#### Health Indicators

##### Symptom Severity

The perceived severity of symptoms was assessed using items from the Patient Health Questionnaire-15, which evaluates 15 somatic symptoms encountered in outpatient settings [33]. It is a widely used brief screening instrument with good psychometric properties for assessing somatic symptoms and screening for somatization [34]. The original scale asks participants to rate how much they were bothered by each symptom in the preceding month. For brevity, we grouped the symptoms under gastrointestinal (ie, stomach pain, constipation, loose bowels, diarrhea, nausea, gas, and indigestion), cardiopulmonary (ie, chest pain, feeling your heart pound or race, and shortness of breath), musculoskeletal (ie, back pain, pain in your arms or legs, and pain in joints like knees and hips), and sleep/energy (ie, headaches, dizziness, fainting spells, feeling tired, having low energy, and trouble sleeping) areas. For each symptom area, the participants evaluated the extent to which they were bothered by any of the symptoms in the preceding 4 weeks. The items were rated on a 5-point Likert scale that ranged from 1 (never) to 5 (always). Higher scores were indicative of more severe symptoms. The internal consistency of the scale was satisfactory (Cronbach α=0.72).

##### Physical Activity

Participants responded to the question—“How many hours a week do you usually exercise to the extent that you sweat and feel shortness of breath?”—to evaluate their physical activity. The response options included (1) less than half an hour per week, (2) about half an hour per week, (3) about 1 hour per week, (4) about 2-3 hours per week, (5) about 4-6 hours per week, and (6) about 7 hours or more per week.

##### BMI

Participants responded to open-ended questions about their height (in centimeters) and weight (in kilograms). The BMI was calculated by weight (kg)/height (m^2^)×10,000 formula.

##### Eating Disorder–Related Risk Propensity

Eating disorder–related risk propensity was assessed with the modified 7-item version [35] of the Eating Disorder Examination Questionnaire [36], a widely used measure of eating pathology. It consists of 3 factors that evaluate dietary restraint, shape/weight overvaluation, and body dissatisfaction. Body dissatisfaction (ie, dissatisfaction with weight and shape) and weight/shape overvaluation (ie, the influence of weight and shape on self-worth) are concerned with the cognitive-evaluative aspects of body image. Dietary restraint assesses the frequency of rigid behaviors to influence weight and shape. The scale is rated with a 7-point forced-choice format that considers the preceding 28 days. Higher scores indicate a greater frequency of dietary restraint (ie, from zero to every day) and a greater severity of body dissatisfaction and weight/shape overvaluation (ie, from “not at all” to “extremely”). The internal consistencies of the factors ranged from 0.89 to 0.91, and the factor loadings and item intercepts were invariant for sex and overweight status in a previous study with adults [35]. The scale’s internal consistency was satisfactory in this study (Cronbach α=0.87).

#### mHealth App Use

App use was determined by asking, “You can use various applications on your phone, tablet, or other mobile devices, like smartwatches. Have you used applications to monitor health and exercise (eg, counting steps, tracking calories, weight, sports activities, eating/drinking, stress, or sleep) in the last year?” The response options were (1) no and (2) yes. mHealth app users responded to an additional question about the frequency of using different types of apps, which included sports (eg, workouts, exercise, running, and strengthening), number of steps, nutrition (eg, calorie intake or expenditure, weight, and nutritional facts), vitals (eg, blood pressure, heart rate, body temperature, and respiratory rate), sleep (eg, sleep patterns), mood and mental well-being (eg, mindfulness, meditation, mental health, and self-esteem), support or control of a diagnosed health condition (eg, diabetes management and depression management), reproductive health (eg, pregnancy, ovulation, menstruation, and sexual health), diagnosis assistance (eg, symptom checking), and emergency care guidance. The response options included (1) never, (2) a few times at most, (3) several times a month, (4) several times a week, (5) every day, and (6) several times a day. The scale’s internal consistency was satisfactory (Cronbach α=0.82).

### Statistical Analysis

Descriptive statistics were run for the sociodemographic characteristics, digital knowledge and use factors, and health indicators. They included means, SDs, and frequencies for the entire sample, app users, and app nonusers. Participants who reported using an mHealth app in the previous year were identified as app users. A multivariate hierarchical binary logistic regression analysis was conducted to examine the characteristics of app users. Sociodemographic factors (age, gender, education, and income) were included in the first step. The second step added the digital knowledge and use factors (eHealth literacy, active and passive use of SNS) and health indicators (perceived symptom severity, physical activity, BMI, and eating disorder–related risk propensity).

The remaining analyses were based on app users’ responses regarding their app use patterns. First, we presented usage frequencies for each app type. Then, we examined the factor structure of app types based on usage frequencies. Guided by the mHealth app categories suggested in the previous literature [4-7], we assumed that mHealth app usage could be divided into two dimensions: mHealth app usage for “promoting wellness” and “managing illness.” We initially ran the exploratory factor analysis (EFA), and based on the factor loading patterns (see the “mHealth_EFA” worksheet in Multimedia Appendix 1), we found that specific mHealth apps (related to nutrition and mental health) appeared to load on two dimensions. Furthermore, the strictly constrained confirmatory factor analysis (CFA) model (see the “mHealth_CFA” worksheet in Multimedia Appendix 1), where, psychometrically speaking, each item is specified to load exclusively on a single factor, yielded an unsatisfactory model fit (comparative fit index=0.86, Tucker-Lewis index=0.81, and root-mean-square error of approximation=0.13). Thus, we subsequently decided to apply the exploratory structural equation modeling (ESEM) method to test this hypothesis. The ESEM used usage frequencies of each app type to determine the underlying factors of use. Although ESEM operates within the traditional CFA framework, it notably allows for the free estimation of cross-loadings while still displaying targeted and constrained factors [37]. This less restrictive approach provides an alternative when CFA does not achieve an adequate model fit. The flexibility of ESEM is advantageous because it more effectively captures the multidimensional aspects of a measure [37,38]. In this study, the ESEM enabled us to assess the model’s fit for our targeted factors (ie, promoting wellness and managing illness). In addition, it could capture the multidimensional aspects of app use by facilitating the cross-loading of app types for both dimensions.

The ESEM approach was also used to predict user characteristics associated with mHealth app usage for our targeted factors (ie, wellness and illness). The dependent variables in the first ESEM model were the frequency of mHealth app usage for promoting wellness and the frequency of mHealth app usage for managing illness. The independent variables were sociodemographics, including age, gender, educational level, and income status. In the second model, we added the predictors of digital knowledge and use factors (eHealth literacy, active and passive use of SNS) and health indicators (symptom severity, physical activity, BMI, eating disorder–related risk propensity).

Lastly, we conducted two separate mediation analyses to test whether digital knowledge, digital use, and health indicators mediated the association between age and app usage. The first mediation analysis examined the relationship between age and wellness app use frequency, and the second examined the relationship between age and illness app use frequency. The analyses were conducted using SPSS software (version 28.0, IBM Corp), the R package “lavaan” (The R Foundation), and the open-source software Jamovi (version 2.6), which provides an R-based graphical user interface. Within Jamovi, the analyses used the R packages “psych,” “lavaan,” and “lm.” Mahalanobis distances were computed to identify multiple outliers, and data from 1.11% (53/4775) of the participants with significant Mahalanobis distance values at *P*<.001 were deleted.

### Ethical Considerations

This study was reviewed and approved by the Research Ethics Committee of Masaryk University (EKV-2023-102). The study complied with ethical standards for human subjects research. Before completing the questionnaires, participants were informed about the purpose of the survey, its anonymity, and their right to refuse participation or skip any question by selecting “I don’t know” or “I prefer not to say.” Written informed consent was obtained before participation. All data were collected and analyzed anonymously, with no personally identifiable information retained. Respondents received monetary compensation for participation, determined by the panel’s standard reward system based on the questionnaire length; the exact amount was not disclosed to the researchers. No images or other materials that could reveal participant identities are included in this manuscript.

## Results

### Data Availability

The sample size consisted of 4775 adults. Of these, data on mHealth app usage were available for 4440 participants and were included in the analyses. Those who did not provide data on mHealth app use (n=282) were more likely to report an older age (t_4720_=17.18, *P*<.001; Cohen *d*=1.055). All *t* values are 2-tailed. There were also significant differences regarding educational level (*χ*^2^_3,4722_=19.5, *P*<.001; *φ*_c_=0.064) and income status (*χ*^2^_3,4722_=117.2, *P*<.001; *φ*_c_=0.158). Data providers were more likely to have a university or higher vocational school education and a monthly income of more than 50,000 CZK than data nonproviders. On the other hand, data nonproviders reported secondary vocational school education more frequently, and they were more likely to have an annual income of less than 20,000 CZK than data providers. No significant gender differences were observed (*χ*^2^_1,4718_=3.04, *P*=.08; *ϕ*=0.025).

### Prevalence of mHealth App Use and Sample Characteristics

Around half of the participants reported using mHealth apps (2172/4440, 48.92%). The characteristics of the total sample, mHealth app users, and nonusers on sociodemographics, digital knowledge and use factors, and health indicators are shown in Table 1.

**Table 1:** Sample characteristics of Czech internet users participating in this cross-sectional study^a^.

Variables					Total sample (N=4440)			App users (n=2172)			App nonusers (n=2268)
**Sociodemographics**											
	Age (years), mean (SD)			44.43 (15.97)			41.36 (15.01)			47.38 (16.31)	
	Gender, woman, n (%)			2185 (49.26)			1108 (51.08)			1077 (47.51)	
	**Educational level, n (%)**										
		None/primary education	286 (6.44)			141 (6.49)			145 (6.39)		
		Secondary vocational	1324 (29.82)			568 (26.15)			756 (33.33)		
		Secondary school	1733 (39.03)			857 (39.46)			876 (38.62)		
		University (or higher vocational)	1097 (24.71)			606 (27.90)			491 (21.65)		
	**Income status, n (%)**										
		Up to 20,000 CZK^b^	454 (10.23)			175 (8.06)			279 (12.30)		
		20,001-35,000 CZK	1364 (30.72)			623 (28.68)			741 (32.67)		
		35,001-50,000 CZK	1455 (32.77)			715 (32.92)			740 (32.63)		
		50,001 CZK and more	1167 (26.28)			659 (30.34)			508 (22.40)		
**Digital knowledge and use, mean (SD)**											
	eHealth literacy			3.73 (0.67)			3.81 (0.65)			3.65 (0.68)	
	Passive SNS use^c^			1.9 (0.94)			2.07 (1)			1.74 (0.83)	
	Active SNS use^d^			1.49 (0.82)			1.59 (0.9)			1.4 (0.73)	
**Health indicators, mean (SD)**											
	Symptom severity			2.55 (0.83)			2.6 (0.81)			2.5 (0.84)	
	Physical activity			2.82 (1.63)			3.10 (1.59)			2.52 (1.62)	
	BMI			27.66 (5.7)			27.49 (5.49)			27.82 (5.9)	
	ED-related risk^e^			2.73 (1.4)			2.97 (1.41)			2.5 (1.34)	

^a^All values are calculated based on available (nonmissing) data; denominators may therefore vary across variables.

^b^A currency exchange rate of 1 CZK=US $0.048 is applicable.

^c^Passive SNS use: passive use of social networking sites for health information.

^d^Active SNS use: active use of social networking sites for health information.

^e^ED-related risk: eating disorder–related risk propensity (measured by body dissatisfaction, dietary restraint, and weight/shape overvaluation).

### Characteristics of mHealth App Users

The multivariate hierarchical binary logistic regression analysis to evaluate the factors associated with mHealth app use is shown in Table 2. Model 1 reports the sociodemographic variables—age, gender, educational level, and income status. Model 2 tests the direct effects of digital knowledge and use factors (eHealth literacy and active and passive use of SNS for health information) and health indicators (symptom severity, physical activity, BMI, and eating disorder–related risk propensity). All models report odds ratios with 95% CIs.

**Table 2:** Sociodemographic, digital, and health-related predictors of mHealth app use based on multivariate hierarchical binary logistic regression analysis in a cross-sectional sample of Czech internet users (n=3923).

Predictors				Model 1^a^					Model 2^b^		
				OR^c^ (95% CI)		*P* value		OR (95% CI)			*P* value
**Sociodemographics**											
	Age (years)			0.98 (0.97-0.98)		<.001		0.98 (0.98-0.99)			<.001
	Gender (reference: male)			1.24 (1.09-1.42)		.001		1.13 (0.98-1.3)			.09
	**Educational level**										
		None/primary education	0.75 (0.56-1.01)		.06		0.8 (0.59-1.09)			.15	
		Secondary vocational	0.87 (0.73-1.04)		.14		0.93 (0.77-1.12)			.44	
		Secondary school	0.92 (0.78-1.08)		.29		0.96 (0.81-1.13)			.61	
		University (or higher vocational)	Reference		Reference		Reference			Reference	
	**Income status**										
		Up to 20,000 CZK^d^	0.56 (0.43-0.71)		<.001		0.54 (041-0.7)			<.001	
		20,001-35,000 CZK	0.78 (0.66-0.93)		<.001		0.78 (0.65-0.93)			.006	
		35,001-50,000 CZK	0.83 (0.7-0.98)		.006		0.83 (0.7-0.99)			.03	
		50,001 CZK and more	Reference		Reference		Reference			Reference	
**Digital knowledge and use**											
	eHealth literacy			—^e^		—		1.17 (1.06-1.3)			.003
	Passive SNS use^f^			—		—		1.35 (1.21-1.51)			<.001
	Active SNS use^g^			—		—		0.91 (0.81-1.02)			.10
**Health indicators**											
	Symptom severity			—		—		1.09 (0.99-1.19)			.07
	Physical activity			—		—		1.18 (1.13-1.23)			<.001
	BMI			—		—		1 (0.99-1.02)			.57
	ED-related risk^h^			—		—		1.18 (1.12-1.25)			<.001

^a^Nagelkerke *R*^2^=0.056.

^b^Nagelkerke *R*^2^=0.128.

^c^OD: odds ratio.

^d^A currency exchange rate of CZK 1=US $0.048 is applicable.

^e^Not applicable.

^f^Passive SNS use: passive use of social networking sites for health information.

^g^Active SNS use: active use of social networking sites for health information.

^h^ED-related risk: eating disorder–related risk propensity (measured by body dissatisfaction, dietary restraint, and weight/shape overvaluation).

The significant predictors of having an app were age, gender, and income status in model 1. Participants who were younger, female, and had a monthly income of more than 50,000 CZK (vs up to 20,000 CZK, between 20,001 and 35,000 CZK, or between 35,001 and 50,000 CZK) were more likely to use mHealth apps. In model 2, digital knowledge, use factors, and health indicators were entered. The role of gender became insignificant when additional variables were included in model 2. After adjusting for the roles of sociodemographic characteristics, the results demonstrated that app users had higher eHealth literacy and were more likely to engage in passive use of SNS for health information. Among the health indicators, higher physical activity and a propensity for risk related to eating disorders were predictive of mHealth app use.

### mHealth App Use by Type of App

The frequency of use for each mHealth app type among app users is shown in Table 3. The most frequently used mHealth app type was the number of steps. The percentage of participants who count steps daily or several times a day was 65.99% (1430/2167). It was followed by mHealth apps to track sleep, vitals, and sports. The percentage of participants who used apps daily or several times a day for monitoring sleep was 31.95% (691/2163), 28.22% (611/2165) for monitoring vitals, and 18.86% (407/2158) for sports. App users were least likely to have ever used apps for emergency care guidance, diagnosis assistance, or to manage diagnosed conditions. The percentages of participants who reported nonuse of apps for emergency care guidance, diagnosis assistance, and diagnosed conditions were 76.72% (1654/2156), 71.62% (1542/2153), and 70.49% (1517/2155), respectively.

**Table 3:** Frequency of using different types of mHealth apps in a cross-sectional sample of Czech internet users (n=2175).

Type of app	Never, n (%)	A few times at most, n (%)	Several times a month, n (%)	Several times a week, n (%)	Daily, n (%)	Several times a day, n (%)
Sports (n=2158)	449 (20.83)	489 (22.66)	394 (18.26)	419 (19.42)	328 (15.2)	79 (3.66)
Number of steps (n=2167)	60 (2.77)	189 (8.72)	224 (10.34)	264 (12.18)	1174 (54.18)	256 (11.81)
Nutrition (n=2161)	803 (37.16)	565 (26.15)	299 (13.84)	219 (10.13)	222 (10.27)	53 (2.45)
Vitals (n=2165)	382 (17.64)	418 (19.31)	392 (18.11)	362 (16.72)	503 (23.23)	108 (4.99)
Sleep (n=2163)	530 (24.5)	408 (18.86)	277 (12.81)	257 (11.88)	644 (29.77)	47 (2.17)
Mood and well-being (n=2156)	1103 (51.16)	447 (20.73)	222 (10.3)	177 (8.21)	180 (8.35)	27 (1.25)
Diagnosed conditions (n=2155)	1517 (70.49)	266 (12.36)	136 (6.32)	104 (4.83)	105 (4.88)	24 (1.12)
Reproductive health (n=2152)	1327 (61.52)	233 (10.8)	314 (14.56)	125 (5.8)	131 (6.07)	27 (1.25)
Diagnosis assistance (n=2153)	1542 (71.62)	323 (15)	134 (6.22)	81 (3.76)	58 (2.69)	15 (0.7)
Emergency care guidance (n=2156)	1654 (76.72)	267 (12.38)	90 (4.17)	78 (3.62)	56 (2.6)	11 (0.51)

### Factor Analysis for mHealth Apps

**Figure 1:**
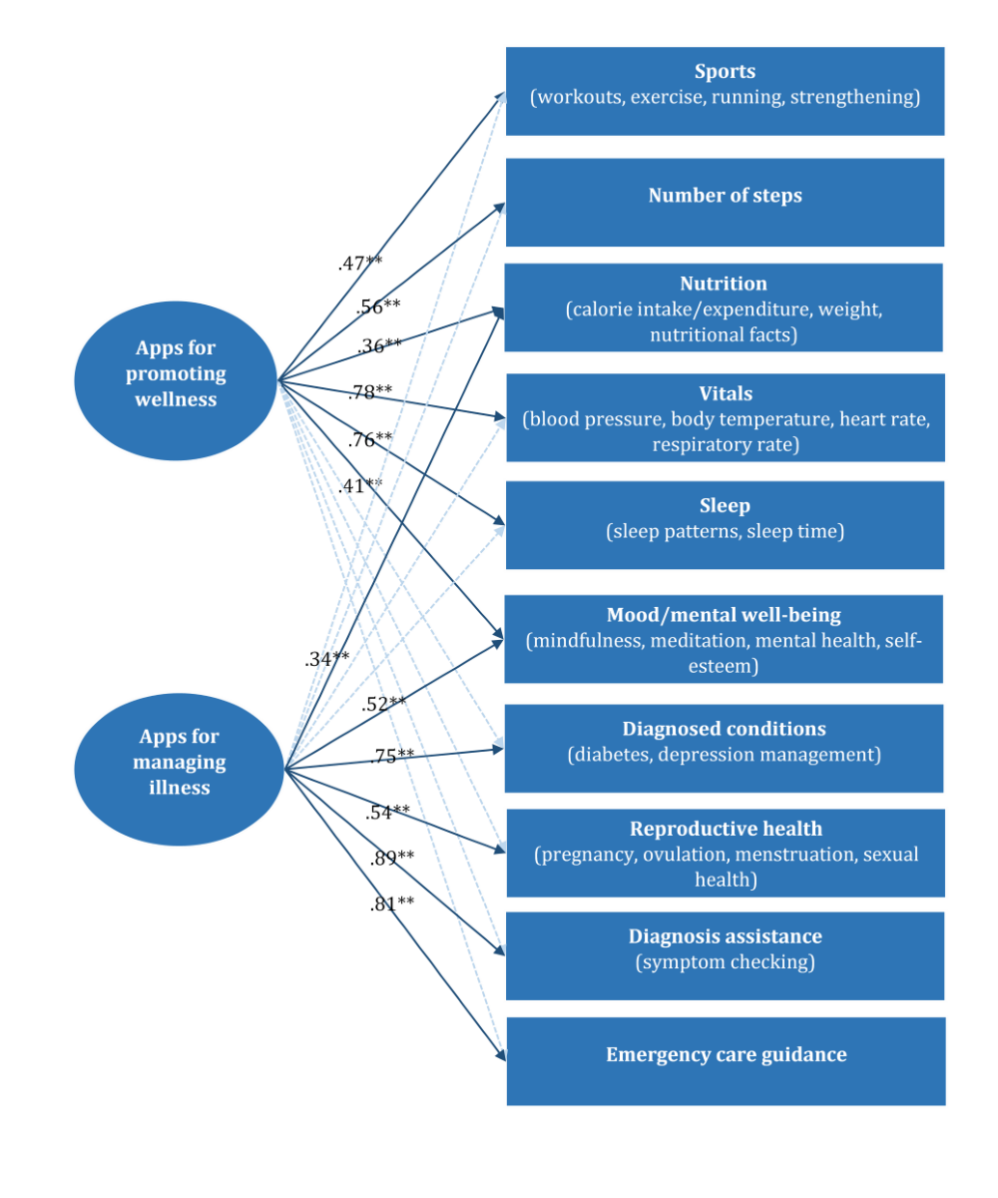
Two-factor structure of mobile health app usage identified using exploratory structural equation modeling in a cross-sectional sample of Czech internet users (n=2152). The numerical values represent factor loadings. **P<.001.

The results confirmed the 2-factor structure of the mHealth app measure with good construct validity (*χ*²_26_=71.9, comparative fit index=0.99, Tucker-Lewis index=0.99, root-mean-square error of approximation=0.03, and standardized root-mean-square residual=0.03). The factor loadings are shown in Figure 1. The results revealed that mHealth usage could be categorized into two dimensions: mHealth app usage for promoting wellness and mHealth app usage for managing illness.

### Characteristics of Wellness and Illness App Users

Table 4 presents the correlations between mHealth app usage and the predictors.

**Table 4:** Spearman correlations between mHealth app use and sociodemographic, digital, and health-related variables in a cross-sectional sample of Czech internet users^a^.

Variables		Wellness apps^b^	Illness apps^c^	Age	Gender	Education	Income	eHealth literacy	SNS active^d^	SNS passive^e^	Sx^f^	BMI	PA^g^	ED risk^h^
**Wellness apps**														
	*ρ* ^i^	1												
	*P* value	—^j^												
	n	—												
**Illness apps**														
	*ρ*	0.645	1											
	*P* value	<.001	—											
	n	2154	—											
**Age**														
	*ρ*	–0.207	–0.27	1										
	*P* value	<.001	<.001	—										
	n	2178	2161	—										
**Gender**														
	*ρ*	0.031	–0.08	0.009	1									
	*P* value	0.15	<.001	0.55	—									
	n	2175	2159	4771	—									
**Education**														
	*ρ*	0.007	–0.078	–0.077	–0.053	1								
	*P* value	0.75	<.001	<.001	<.001	—								
	n	2178	2161	4775	4771	—								
**Income**														
	*ρ*	0.043	–0.042	–0.169	0.148	0.273	1							
	*P* value	0.05	0.05	<.001	<.001	<.001	—							
	n	2178	2161	4775	4771	4775	—							
**eHealth literacy**														
	*ρ*	0.123	0.086	–0.193	0.006	0.183	0.155	1						
	*P* value	<.001	<.001	<.001	0.69	<.001	<.001	—						
	n	2174	2157	4756	4752	4756	4756	—						
**SNS active**														
	*ρ*	0.202	0.47	–0.298	–0.07	–0.057	–0.051	0.1	1					
	*P* value	<.001	<.001	<.001	<.001	<.001	<.001	<.001	—					
	n	2164	2147	4732	4728	4732	4732	4715	—					
**SNS passive**														
	*ρ*	0.223	0.462	–0.243	–0.155	–0.012	–0.04	0.165	0.668	1				
	*P* value	<.001	<.001	<.001	<.001	0.4	0.005	<.001	<.001	—				
	n	2165	2148	4740	4736	4740	4740	4723	4731	—				
**Sx**														
	*ρ*	0.099	0.242	0.009	–0.146	–0.106	–0.147	–0.032	0.245	0.304	1			
	*P* value	<.001	<.001	0.52	<.001	<.001	<.001	0.03	<.001	<.001	—			
	n	2178	2161	4766	4762	4766	4766	4747	4724	4732	—			
**BMI**														
	*ρ*	0.005	–0.047	0.279	0.105	–0.112	–0.019	–0.085	–0.06	–0.057	0.086	1		
	*P* value	0.83	0.03	<.001	<.001	<.001	0.2	<.001	<.001	<.001	<.001	—		
	n	2178	2161	4775	4771	4775	4775	4756	4732	4740	4764	—		
**PA**														
	*ρ*	0.179	0.093	–0.19	0.072	0.13	0.128	0.16	0.088	0.108	–0.055	–0.137	1	
	*P* value	<.001	<.001	<.001	<.001	<.001	<.001	<.001	<.001	<.001	<.001	<.001	—	
	n	2034	2019	4299	4295	4299	4299	4283	4259	4265	4295	4299	—	
**ED risk**														
	*ρ*	0.199	0.307	–0.116	–0.14	0.02	–0.009	0.053	0.227	0.296	0.307	0.276	0.154	1
	*P* value	<.001	<.001	<.001	<.001	0.18	0.52	<.001	<.001	<.001	<.001	<.001	<.001	—
	n	2164	2147	4731	4727	4731	4731	4712	4689	4697	4726	4731	4273	—

^a^Sample sizes vary by correlation pair; Ns are shown within the table.

^b^Wellness apps: frequency of using mHealth apps for promoting wellness.

^c^Illness apps: frequency of using mHealth apps for managing illness.

^d^SNS active: active use of social networking sites (SNS) for health information.

^e^SNS passive: passive use of social networking sites (SNS) for health information.

^f^Sx: perceived severity of symptoms.

^g^PA: physical activity.

^h^ED risk: eating disorder–related risk propensity (measured by body dissatisfaction, dietary restraint, and weight/shape overvaluation).

^i^*ρ*: Spearman rank order correlation.

^j^Not applicable.

Aligned with the ESEM method for factor analysis, the current model did not restrict the factor loadings of mHealth apps to a particular factor. Table 5 shows the results. When interpreting the results, we focused not only on statistical significance but also on the effect sizes. Following widely acknowledged guidelines, standardized path coefficients (β) between 0.1 and 0.3 were interpreted as indicating a small effect, those between 0.3 and 0.5 as medium, and those above 0.5 as large effects [39]. In the first model, which included only sociodemographic independent variables, app use to promote wellness was predicted by age (standardized β=–0.22, *P*<.001), indicating that older age was correlated with less mHealth app use to promote wellness. Except for gender, sociodemographic characteristics, including age, educational level, and income status, were significantly linked to app use in managing illness. However, the effect size of income was negligible (retaining three decimal places, standardized β=0.097). Likewise, the effect of education on illness-related app use was also at the boundary of a negligible effect size (retaining three decimal places, standardized β=–0.106). Furthermore, after including health-, digital knowledge-, and use-related variables, none of the sociodemographic variables remained significant. We did not interpret them further because both education level and income were already at the boundary of a negligible effect size when only sociodemographic variables were considered, and adding additional variables merely pushed them fully into the negligible range. Notably, however, the reduction in effect size of age was considerable for both app types, which prompted us to conduct further analyses to examine the mediating roles of digital knowledge, digital use, and health indicators in the association between age and wellness- and illness-related app use.

**Table 5:** Predictors of the frequency of using mHealth apps to promote wellness and manage illness based on exploratory structural equation modeling in a cross-sectional sample of Czech internet users^a^.

		Model 1 (n=2152)				Model 2 (n=1985)		
Predictors		Standardized β	SE	*P* value	Standardized β		SE	*P* value
**Apps to promote wellness**								
	Age	–0.22	0.00	<.001	–0.18		0.00	<.001
	Gender	0.03	0.03	.07	0.04		0.05	.04
	Education	0.03	0.02	.15	0.02		0.03	.24
	Income	0.02	0.02	.27	0.02		0.02	.42
	eHealth literacy	—^b^	—	—	0.10		0.02	<.001
	Active SNS use^c^	—	—	—	0.07		0.04	.12
	Passive SNS use^d^	—	—	—	0.08		0.05	.07
	Symptom severity	—	—	—	0.04		0.03	.08
	BMI	—	—	—	0.08		0.03	<.001
	Physical activity	—	—	—	0.15		0.02	<.001
	ED-related risk^e^	—	—	—	0.09		0.03	<.001
**Apps to manage illness**								
	Age	–0.23	0.00	<.001	–0.04		0.00	.07
	Gender	0.02	0.03	.31	0.01		0.08	.75
	Education	–0.11	0.02	<.001	–0.05		0.04	.05
	Income	–0.10	0.02	<.001	–0.02		0.04	.40
	eHealth literacy	—	—	—	0.00		0.04	.93
	Active SNS use^c^	—	—	—	0.62		0.15	<.001
	Passive SNS use^d^	—	—	—	0.06		0.11	.49
	Symptom severity	—	—	—	0.04		0.04	.20
	BMI	—	—	—	–0.07		0.04	.01
	Physical activity	—	—	—	0.02		0.03	.46
	ED-related risk^e^	—	—	—	0.11		0.04	<.001

^a^Sample sizes vary across models; Ns are reported in the table.

^b^Not applicable.

^c^SNS active: active use of social networking sites for health information.

^d^SNS passive: passive use of social networking sites for health information.

^e^ED-related risk: eating disorder–related risk propensity (measured by body dissatisfaction, dietary restraint, and weight/shape overvaluation).

The results also suggested that eHealth literacy and physical activity were significantly correlated with the frequency of mHealth app usage to promote wellness. Regarding the use of mHealth apps to manage illness, the active use of SNS for health-related information and eating disorder–related risk propensity were significant predictors of the frequency of use.

For a more robust interpretation, we additionally performed basic linear regression analyses using the ordinary least squares method (see Table 6). The standardized estimates did not differ substantially from the main analyses, except that active use of SNS for health-related information (SNS active) was significant in the simple linear regression model but was not significantly associated with mHealth app usage for promoting wellness in the full model. It is worth noting, however, that the effect size was small (standardized β=0.13, *P*<.001). Similarly, in the ESEM model, the effect size of eating disorder risk on wellness-related mHealth app usage was negligible (standardized β=0.09), and in the ordinary least squares regression model, it was 0.12—still very small, bordering on a negligible effect size. Such a minor difference cannot be considered meaningful. Considering that the ESEM model accounted for factor loadings and that the effect size in the simple regression remained small, we regarded this discrepancy as acceptable. Therefore, the results from the ESEM model (Table 5) should be prioritized for interpretation.

**Table 6:** Predictors of the frequency of using mHealth apps to promote wellness and manage illness based on simple regression models in a cross-sectional sample of Czech internet users^a^.

					Model 1							Model 2				
Predictors					Standardized β		SE		*P* value		Standardized β			SE		*P* value
**Apps to promote wellness (n=2005)**																
	Age				–0.22		0.00		<.001		–0.15			0.00		<.001
	Gender (2-1)^b^				0.07		0.04		.08		0.10			0.04		.02
	**Education**															
		3-4^c^			–0.01		0.05		.91		0.01			0.05		.81
		2-4^d^			0.05		0.06		.41		0.05			0.06		.37
		1-4^e^			–0.12		0.10		.23		–0.13			0.09		.18
	Income				0.00		0.02		.88		0.02			0.02		.48
	eHealth literacy				—^f^		—		—		0.08			0.03		<.001
	Active SNS use^g^				—		—		—		0.13			0.03		<.001
	Passive SNS use^h^				—		—		—		0.09			0.03		.004
	Symptom severity				—		—		—		0.04			0.03		.12
	BMI				—		—		—		0.04			0.00		.12
	Physical activity				—		—		—		0.15			0.01		<.001
	ED-related risk^i^				—		—		—		0.12			0.02		<.001
**Apps to manage illness** **(n=1991)**																
	Age				–0.26		0.00		<.001		–0.09			0.00		<.001
	Gender (2-1)^b^				–0.02		0.04		.64		0.00			0.03		.85
	**Education**															
			3-4^c^	0.03		0.05		.62		0.02			0.04		.62	
			2-4^d^	0.26		0.06		<.001		0.14			0.05		.004	
			1-4^e^	0.13		0.09		.17		0.02			0.07		.84	
	Income				–0.08		0.02		<.001		–0.01			0.02		.50
	eHealth literacy				—		—		—		0.02			0.02		.19
	Active SNS use^g^				—		—		—		0.51			0.02		<.001
	Passive SNS use^h^				—		—		—		0.07			0.02		.01
	Symptom severity				—		—		—		0.04			0.02		.05
	BMI				—		—		—		–0.04			0.00		.03
	Physical activity				—		—		—		0.05			0.01		.003
	ED-related risk^i^				—		—		—		0.14			0.01		<.001

^a^Sample sizes vary across models; Ns are reported in the table.

^b^2-1: male-female.

^c^3-4: secondary school – university (or higher vocational school).

^d^2-4: secondary vocational school – university (or higher vocational school).

^e^1-4: none/primary education – university (or higher vocational school).

^f^Not applicable.

^g^SNS active: active use of social networking sites for health information.

^h^SNS passive: passive use of social networking sites for health information.

^i^ED-related risk: eating disorder–related risk propensity (measured by body dissatisfaction, dietary restraint, and weight/shape overvaluation).

### Mediation Analysis

**Figure 2:**
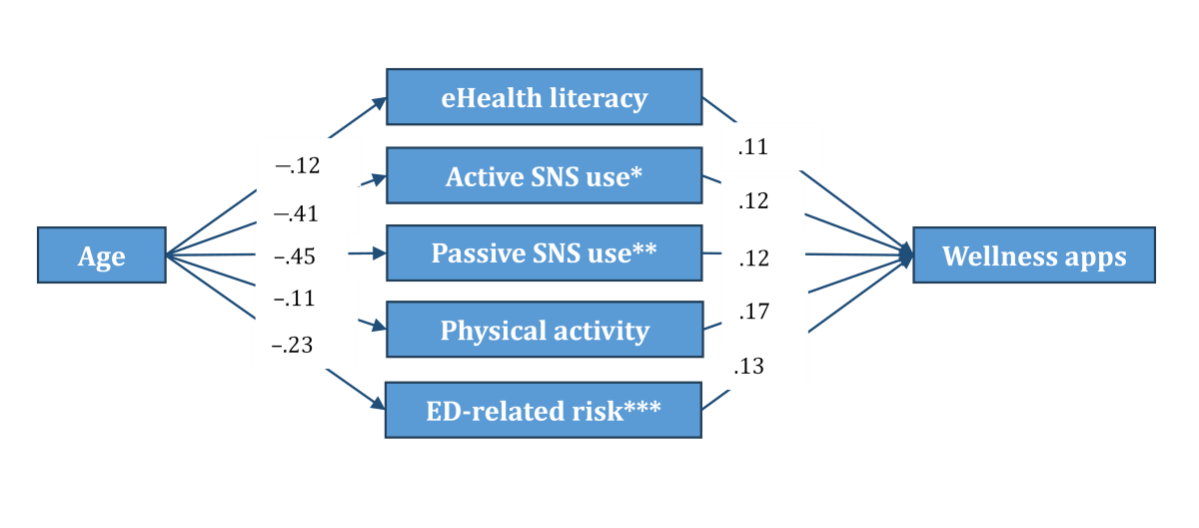
Mediation model depicting associations between age and wellness-related mobile health app use based on exploratory structural equation modeling in a cross-sectional sample of Czech internet users (n=1985). Only statistically significant paths (*P*<.05) are shown. Standardized coefficients are displayed. *SNS active: active use of social networking sites for health information. **SNS passive: passive use of social networking sites for health information. ***ED-related risk: eating disorder–related risk propensity (measured by body dissatisfaction, dietary restraint, and weight/shape overvaluation).

**Figure 3:**
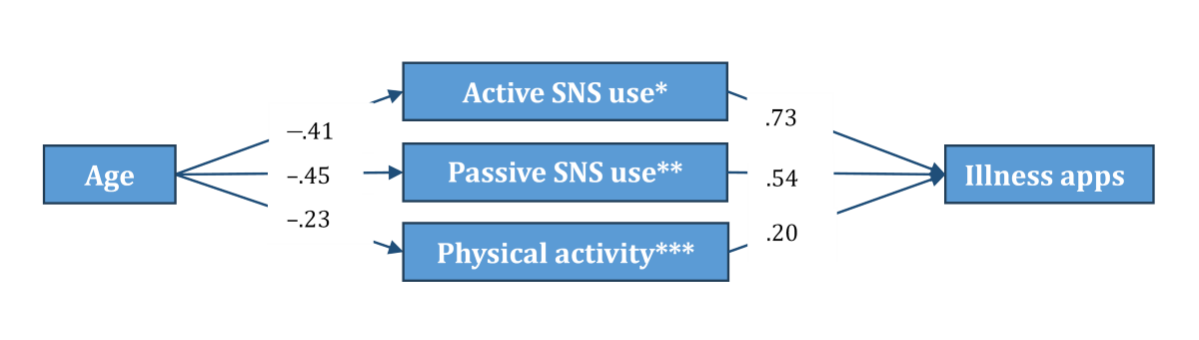
Mediation model depicting associations between age and illness-related mobile health app use based on exploratory structural equation modeling in a cross-sectional sample of Czech internet users (n=1985). Only statistically significant paths (*P*<.05) are shown. Standardized coefficients are displayed. *SNS active: active use of social networking sites for health information. **SNS passive: passive use of social networking sites for health information. ***ED-related risk: eating disorder–related risk propensity (measured by body dissatisfaction, dietary restraint, and weight/shape overvaluation).

Due to the decrease in the effect size of age on two dependent variables after the addition of digital knowledge, digital use, and health indicators, it was worthwhile to explore further whether these variables mediated the relationship between age and mHealth usage. The mediation analysis suggested that older age correlated with more frequent use of wellness apps through “eHealth literacy,” “active SNS use,” “passive SNS use,” “physical activity,” and “eating disorder–related risk propensity” (see Figure 2). On the other hand, “active SNS use,” “passive SNS use,” and “eating disorder–related risk propensity” mediated the relationship between age and frequency of mHealth app use to manage illness (see Figure 3). It is worth noting that the correlations between active and passive SNS use and illness-related mHealth app usage were more robust than those of wellness-related mHealth app usage.

## Discussion

### Overview

This study evaluated the prevalence, user characteristics, and usage patterns of various mHealth apps in a representative sample of Czech adults with internet access. Furthermore, it distinguished between wellness and illness apps using a data-driven approach and identified user characteristics associated with the frequency of use of each dimension. This study provided significant insights into the relationship between user characteristics, intentions, and engagement with mHealth apps.

### Principal Results

#### Prevalence of mHealth App Use

Around half of the participants with internet access (48.92%) had been using mHealth apps. Previous studies with adults have reported prevalence rates ranging from 34.1% to 56.6% in the United States [9-12]. Lower rates were reported in European and Far Eastern countries. For instance, 20.5% of German smartphone owners in 2015 [18] and 24.1% of Hong Kongian smartphone or tablet owners in 2016 [23] used mHealth apps in population-based samples. More up-to-date findings are needed for a more accurate comparison of the prevalence rates across countries.

#### Characteristics of mHealth App Users

##### Sociodemographic Characteristics

Age was significantly associated with using mHealth apps. In line with earlier studies [8], younger adults were more likely to use them. This finding highlights that older adults are underserved in terms of mHealth app use. Lack of awareness, lack of motivation, low self-efficacy, mistrust of technology, and the absence of required technical skills are frequently reported barriers to using mHealth apps among older adults [40,41]. Additionally, cognitive impairment, reduced physical ability, and visual changes associated with aging can compromise the usability of app interfaces in this population [42-45]. Dispositional, attitudinal, and aging-related factors must be considered when addressing this population via apps.

Participants with monthly incomes exceeding 50,000 CZK were more likely to have mHealth apps than those in other income categories (ie, up to 20,000 CZK, between 20,001 and 35,000 CZK, and between 35,001 and 50,000 CZK). This finding is consistent with previous studies that have reported higher annual income as a significant predictor of mHealth app adoption and usage [9,11,12,23]. Higher-income status may facilitate access to apps that require payment to download and use, whereas such apps may be less affordable for low-income individuals. The association between educational level and mHealth app usage was not statistically significant. This finding contrasts with previous studies conducted in the United States [9-12] and Hong Kong [23], but it is similar to an earlier study conducted in Germany [18]. Future studies will clarify whether educational background will influence the adoption and use of apps as they become more affordable, user-friendly, and accessible.

##### Digital Knowledge and Use Factors

Adults with higher eHealth literacy were more likely to have mHealth apps. This finding aligns with previous studies [14,46] and confirms that eHealth literacy is a crucial digital skill for understanding mHealth app use behaviors. Individuals with higher eHealth literacy tend to seek online health information more frequently [47], are more health information-oriented [48], and place greater importance on health-related information [49]. They are also more likely to report competence in self-care [50] and their intention to promote health [51]. These findings underscore that perceived digital skills in finding, understanding, evaluating, and applying online health information in a health context might be precursors to having and using apps.

This study provided initial evidence in support of the association between SNS activity for health information and having mHealth apps. We found that passive use (ie, reading, watching, or following health content) was significantly associated with the use of apps. However, the association between active use (ie, creating, sharing, and commenting on health content) and app use was insignificant. Our results reveal that adopting mHealth apps might be more closely related to consuming health-related information on SNS (ie, passive use) than engaging with online networks for health-related information (ie, active use). This finding aligns with a previous study that reported a significant association between health information-seeking behaviors on social media and the use of mHealth apps for health information [52]. Health information–oriented individuals may use SNS and mHealth apps in a complementary manner to meet their health information needs. Additionally, SNS provides a platform for promoting various functions of mHealth apps [53], which may help meet the personal health care needs of consumers and contribute to the adoption of mHealth apps. These findings suggest that SNS could be a significant promotional channel.

##### Health Indicators

In line with previous research [10,18,23], participants with higher physical activity were more likely to have mHealth apps, which provides further evidence for the association between a physically active lifestyle and mHealth app adoption. We found a significant association between eating disorder–related risks and mHealth app use. Participants who reported higher dietary restraint, weight and shape overvaluation, and body dissatisfaction were more likely to have and use mHealth apps. Prior studies reporting an association between eating disorder–related risks and app usage have primarily focused on apps that promote weight loss and physical activity [28-30]. This study shows that body dissatisfaction, weight and shape overvaluation, and restrictive eating behaviors correlate with overall app adoption.

BMI was not associated with using apps, contrasting with earlier studies that reported a significant association between app usage and a higher BMI [10,18,25]. It is possible that eating disorder–related risks (ie, body dissatisfaction, weight/shape overvaluation, and dieting behaviors) capture app usage behaviors better than body weight. We did not observe a significant association between the use of apps and symptom severity. This finding aligns with studies that found no significant association between health status and mHealth app usage [10-12]. This study focused on a detailed measurement of perceived health status by assessing somatic symptom severity in multiple domains.

### mHealth App Use by Type of App

Apps that count the number of steps were most frequently used. This finding aligns with global statistics, which indicate that the most downloaded app in 2022 was a step counter [54]. One reason for the frequent use of step counter apps could be related to their simplicity. Previous research showed this to be a significant factor in determining the frequency of using apps for physical activity [25]. Apps that count the number of steps may take less effort from consumers to integrate them into everyday life. These apps are often built-in apps delivered in the latest releases of major smartphone companies, and their availability could enhance increased adoption and use. Apps that track sleep, vitals, and sports activities were also popular. In a recent qualitative study, app users reported that their continued use of apps with tracking functions (eg, tracking steps, exercise, heart rate, and sleep) was associated with the perceived usefulness of these apps for maintaining and improving their health and well-being [55].

### Factor Analysis for mHealth Apps

Using a data-driven approach with ESEM, we confirmed the 2-factor structure of mHealth app usage in the analyses. The apps under the “promoting wellness” factor were related to lifestyle behaviors (eg, sports apps, nutrition apps, and number of steps apps), monitoring of health indicators (eg, vitals apps and sleep apps), and the enhancement of mental vitality (eg, mood and mental well-being apps). This factor consisted of apps focusing on preventive health behaviors to promote wellness and reduce the risk of illness. The apps under the “managing illness” factor focused on maintaining health (eg, apps for reproductive health and diagnosis assistance) and providing support for illnesses (eg, apps for diagnosed conditions and emergency care guidance). We found that nutrition apps and mood and mental well-being apps cross-loaded on both factors. Several health conditions (eg, diabetes) require close monitoring of nutritional intake [56]. Therefore, it is reasonable that app users might monitor nutrition not only for promoting wellness but also for managing diagnosed health conditions. Similarly, mood and mental well-being are broad domains, and app users’ intentions may have dual relevance, encompassing both the promotion of mood and mental well-being for wellness purposes and the management of mental health problems as part of a treatment regimen [57]. In conclusion, the factor structure of our mHealth measure was feasible. It might guide future research studies on app usage for health promotion and illness management.

### Characteristics of Wellness and Illness App Users

Age was significantly associated with the frequency of using wellness apps. Older adults used them less frequently to promote wellness. This finding is disappointing because older adults are more susceptible to chronic conditions like diabetes, hypertension, and cardiovascular disease [58]. Effective characteristics of wellness apps that enhance the adherence of older adults should be identified to combat their digital exclusion.

eHealth literacy was significantly associated with more frequent use of apps to promote wellness, but no such association was observed for apps that manage illness. Previous research has confirmed that higher eHealth literacy is associated with perceiving mHealth apps as effective and using them more frequently for health-related behavioral change [14,15,45]. Our findings indicate that this association might be particularly relevant for behaviors that promote health. Individuals with higher eHealth literacy are more motivated to engage in behaviors to regulate their diet, sleep, and physical activity [59]. They are more likely to adhere to recommendations to reduce the risk of illness [60] and to manage stress [51]. Having the necessary digital skills to navigate within apps might reinforce the regular use of wellness apps in this population, which is inherently more motivated to engage in health-promoting behaviors.

We found a significant association between active SNS use for health information and the more frequent usage of apps to manage illness. This finding suggests that proactive engagement with online social networks for health information plays a crucial role in the frequent use of illness apps. A recent study reported a significant association between having a friend to discuss health-related issues and the use of mHealth apps [12]. We consider that our finding aligns with this previous study because the active use of SNS promises a platform for individuals to interact with their close networks about health and connect with others who might have similar health concerns. Users of illness apps might use SNS complementarily to exchange health information, share experiences, discuss health-related decisions, and provide and receive support from their online social networks [61]. The active use of these networks can synergistically encourage compliance with apps to manage illness, as they offer real-time feedback and a direct line of communication to those who may face similar health concerns.

Higher physical activity was associated with a more frequent use of wellness apps. Wellness apps (eg, Fitbit) offer a wide range of functions that physically active individuals can use to monitor and regulate their physical activity, conditioning, and fitness [62]. They also offer a platform to manage their diet, vitals, energy levels, and mental state [62], which addresses well-being holistically. These apps may be particularly appealing to physically active adults who may be inherently more motivated to pursue a healthy lifestyle [63,64] and thus use them more regularly to enhance their well-being.

This study provided initial evidence to indicate that individuals with elevated risks of eating disorders use mHealth apps more frequently to manage illness. Body image concerns and dieting behaviors may be influenced by various health conditions beyond eating disorders (eg, obesity, diabetes, and depression) [65,66]. The patterns and consequences of using apps can differ substantially depending on the health domain for which they are used. Previous studies focused on weight loss and physical activity apps to examine the association between unintended uses and the relative consequences of using these apps in exacerbating or triggering adverse outcomes among individuals with body image concerns and dieting behaviors [67-69]. These previous findings underscored that underweight BMI goals had a detrimental impact on eating disorder–related symptoms and behaviors, such as more restrictive eating behaviors, control, and obsession with eating and exercise. In contrast, more realistic goals regarding weight and shape, along with motivation toward healthy lifestyle choices, were associated with improved food choices, exercise behaviors, and health outcomes. Therefore, future research should specify health behavior goals and the consequences of using apps to manage illness in individuals with elevated body image and eating concerns.

### Mediation Analysis

As mentioned earlier, while conducting the analyses, we observed that the effect size of age was considerably reduced for both app types after including digital knowledge, digital use, and health-related variables in the equation. Therefore, although it was not among our initial research aims, we decided to explore whether any of these variables mediated the association between age and app usage. The results revealed important insights into the association between older age and the less frequent use of apps for wellness and illness purposes.

The association between age and mHealth app use was negatively mediated by the active and passive use of SNS for health information. In other words, older adults were less likely to use SNS to seek health information (ie, passive use) and to interact with online networks about health (ie, active use), which was significantly associated with less frequent app use. This association was significant for both app types and notably stronger for apps to manage illness. Compared with younger adults, older adults use social media and search engines less frequently for health information [52]. They are also more likely to prefer traditional media (eg, television and newspaper) [70]. Our findings indicate that a lack of orientation toward seeking and exchanging health information on SNS could hinder older adults’ use of mHealth apps. SNS may provide know-how about app functions and facilitate social support for sustained use of mHealth apps.

The association between age and wellness app use was negatively mediated by eHealth literacy. Older adults had lower eHealth literacy, which was significantly associated with less frequent use of wellness apps. Previous studies have confirmed the associations between older age, lower eHealth literacy, and the use of mHealth apps [8,71]. Our finding shows that enhancing eHealth literacy skills could significantly increase app compliance for health behaviors that promote well-being in this population.

The association between age and wellness app use was negatively mediated by physical activity. Older adults reported lower physical activity levels, which were significantly associated with less frequent use of wellness apps. Older age is characterized by reduced physical activity [72], and older adults are less likely to adhere to recommendations for physical exercise [73,74]. However, a physically active lifestyle is associated with a reduced risk of all-cause mortality, a better quality of life, and improved cognitive functioning in older age [75]. Wellness apps offer novel opportunities to promote physical activity, reduce sedentary behaviors, and enhance physical and mental well-being in this population [76]. Thus, our finding highlights a significant gap in their dissemination to older adults, who might need such strategies most. The obstacles associated with lower acceptance and the usage of wellness apps and strategies to empower older adults’ self-efficacy regarding healthy aging should be identified.

Finally, the association between age and mHealth app use was negatively mediated by eating disorder–related risk propensity. This association was significant for both app types. In other words, older adults were less likely to report body image concerns and dieting behaviors, which, in turn, were significantly associated with less frequent use of apps for wellness and illness purposes. The importance placed on physical appearance is reduced by increasing age. Body dissatisfaction, weight and shape overvaluation, and dieting behaviors are reported less frequently after the age of 40 [77]. In general, lower body image preoccupation is associated with healthier lifestyle behaviors, psychological adjustment, and better management of health conditions that require close monitoring of health indicators and dietary behaviors [65,78,79]. Therefore, it is interesting to find that a lower eating disorder–related risk is associated with less frequent use of apps in older adults. Further research is needed to understand the body image perceptions, dieting behaviors, and weight- and shape-related concerns of older adults, as well as their associations with the use of apps.

### Limitations and Future Research

The findings of this study should be interpreted in light of its limitations. Due to the cross-sectional study design, temporal and causal associations between the variables could not be assessed. For instance, we cannot ascertain whether eating disorder–related risks determine mHealth app use or whether they are exacerbated due to the use of mHealth apps. Our mHealth app measure consisted of apps frequently downloaded in app stores for health purposes. However, it is not exhaustive and can be improved in later studies. We used a data-driven approach (ESEM) to categorize app usage into wellness and illness dimensions. This approach enabled us to demonstrate the multidimensional structure of mHealth app usage. Wellness and illness dimensions also drew parallels between preventive health behaviors and illness behaviors in the health context [80]. Although ESEM allows for more flexible estimation of latent constructs compared with traditional CFA, its results can still be sensitive to model specification and sample characteristics. Thus, despite ESEM being a better option in this study, the findings should be interpreted with caution, and future studies are encouraged to replicate the model using different samples or model structures.

We determined the usage patterns of apps based on their frequency of use. This approach limited our ability to identify the healthy and unhealthy uses of apps. For instance, excessive use of sports apps can be dysfunctional when users engage with them to the point of overexercising. Weight loss apps might have adverse consequences for at-risk individuals if they are used frequently due to an obsession with eating and body image concerns.

The assessments were based on self-reports. Thus, we could not control the response characteristics of the participants. We used adapted versions of frequently used questionnaires to assess symptom severity and SNS use for health information. Additionally, eating disorder–related risk propensity and eHealth literacy measures have not been previously adequately validated in the Czech context. Nevertheless, all the measures had adequate internal consistency in this study. Besides, we conducted cognitive testing with a subsample of participants to ensure the comprehensibility of items and the quality of the assessment procedure.

Future research is needed to investigate the health behaviors and outcomes associated with the use of apps. Usage intentions and motivations are essential to understanding the healthy and unhealthy uses of mHealth apps. Therefore, future studies should consider the health behavior goals associated with using apps and identify the factors that contribute to unintended uses and consequences.

### Conclusions

This study demonstrates that sociodemographic, digital, and health-related factors might jointly shape engagement with mHealth apps. eHealth literacy and health-related SNS activity emerged as key digital correlates of use, highlighting the potential of promoting digital inclusion to support equitable participation in digital health. Eating disorder–related risk propensity—reflecting body image concerns and dietary restraint—was associated with the overall app adoption and frequent use of apps to manage illness, underscoring the importance of examining app use purposes and health outcomes among individuals with elevated body image and dietary concerns, and the need for future research and app development to promote health-supportive use while minimizing appearance-driven behaviors. Indirect associations of age through eHealth literacy, health-related SNS use, physical activity, and eating disorder–related risk further highlight multidimensional barriers older adults face in adopting and maintaining mHealth app use. Collectively, these findings indicate that mHealth engagement reflects broader social, digital, and psychological inequalities rather than individual preferences alone. Enhancing digital skills, promoting inclusive app design, and addressing body image- and diet-related use may foster equitable mHealth participation and help prevent widening health disparities across age and user groups.

## Data Availability

The data and the analysis scripts are shared via the Open Science Framework [81].

## Appendix

Multimedia Appendix 1 Additional analysis of mobile health app usage.

## Abbreviations

CFA: confirmatory factor analysis
EFA: exploratory factor analysis
ESEM: exploratory structural equation modeling
mHealth: mobile health
NUTS3: Nomenclature of Territorial Units for Statistics
OR: odds ratio
SNS: social networking site
STROBE: Strengthening the Reporting of Observational Studies in Epidemiology
